# Imaging observations of pulmonary inflammatory myofibroblastic tumors in patients over 40 years old

**DOI:** 10.3892/ol.2015.2923

**Published:** 2015-02-02

**Authors:** JIANG WU, HONG ZHU, KAI LI, CAI-YUN YUAN, YAN-FEN WANG, GUANG-MING LU

**Affiliations:** 1 Department of Nuclear Medicine, Jinling Hospital, School of Medicine, Nanjing University, Nanjing, Jiangsu 210002, P.R. China; 2 Department of Pharmacology, Soochow University, Suzhou, Jiangsu 215123, P.R. China; 3 Department of Medical Imaging, Jinling Hospital, School of Medicine, Nanjing University, Nanjing, Jiangsu 210002, P.R. China; 4 Department of Pathology, Jinling Hospital, School of Medicine, Nanjing University, Nanjing, Jiangsu 210002, P.R. China

**Keywords:** pulmonary inflammatory myofibroblastic tumor, computed tomography, positron emission tomography, single photon emission computed tomography

## Abstract

Pulmonary inflammatory myofibroblastic tumors (PIMTs) are extremely rare in adults. If occurring in patients >40 years old, PIMT should be rapidly distinguished from lung cancer. The present study aimed to characterize the imaging features of PIMT in patients >40 years old in order to improve the diagnosis of PIMT. The imaging data of 10 patients with PIMT were reviewed retrospectively. Of the patients, eight underwent computed tomography (CT), two underwent positron emission tomography (PET)/CT and four underwent single-photon emission computed tomography (SPECT). Unenhanced CT revealed 10 lesions with a maximum diameter ranging between 5 and 57 mm located in the lower (n=6) or upper (n=4) lobe, in a peripheral (n=9) or central (n=1) region, and that were well- (n=4) or ill-defined (n=6), and round to oval (n=5) or irregular (n=5) in shape. Calcification (n=3), necrosis (n=6), cavity (n=4), air bronchogram (n=6) and obstructive pneumonia (n=1) were also observed in the patients. Contrast-enhanced CT revealed six lesions with moderate to high contrast enhancement in the arterial and venous phases, including four lesions with delayed enhancement. PET/CT identified two lesions with increased tracer uptake that were homogeneous and heterogeneous and each exhibited a maximal standard uptake value (SUV_max_) of 6.0 and 5.4, respectively. The delayed PET/CT revealed foci that each exhibited an increased SUV_max_ of 6.9 and 5.9, respectively. SPECT demonstrated no definitive bone metastases, but did reveal atypical hypertrophic pulmonary osteoarthropathy in one patient. The combined imaging methods may lead to a more precise evaluation of PIMT in patients >40 years old.

## Introduction

Inflammatory myofibroblastic tumor (IMT) is a rare disorder that was previously referred to by a variety of synonyms, including inflammatory pseudotumor, plasma cell granuloma, fibroxanthoma, fibrous histiocytoma and xanthogranuloma (1). In 2002, the World Health Organization classification scheme defined IMT as a ‘distinctive lesion composed of myofibroblastic spindle cells accompanied by an inflammatory infiltrate of plasma cells, lymphocytes and eosinophils’ (2). Despite its precise definition, IMT remains controversial in regard to its nature and origin. With alterations in the anaplastic lymphoma kinase (ALK) gene and an overexpression of ALK protein reported in many cases of IMT, the concept that IMT is a true neoplasm, rather than a reactive process, has been increasingly accepted (2–4). Furthermore, reported cases of IMTs exhibiting aggressive growth, local invasion, recurrence and even distant metastasis, also supports this concept (5–10).

IMT may occur at any age, but usually affects children and adults <40 years old (6,11). IMT primarily affects the lung, and accounts for 0.04–1% of all reported lung tumors (12,13). Pulmonary IMT (PIMT) is the most frequently diagnosed primary lung mass in children (14). Therefore, a large number of studies regarding childhood PIMT exist in the literature. For adults, particularly those >40 years old, a relatively small number of studies concerning PIMT have been published, due to its rarity. Furthermore, the most common malignant tumor of the lung, lung cancer, predominantly affects this age group. Therefore, it is of great importance to identify PIMT in patients over the age of 40.

PIMT is challenging to diagnose in the absence of any pathological evidence, as few of its clinical manifestations and laboratory results are specific. Imaging features of PIMT remain poorly recognized, although a number of radiological studies referring to chest X-ray, computed tomography (CT) and magnetic resonance imaging have been published (12,15,16). Recently, ^18^F-fluorodeoxyglucose (FDG) positron emission tomography (PET)/CT has been widely used for the diagnosis and differential diagnosis of lung masses. Therefore, a requirement exists to extend the understanding of ^18^F-FDG PET/CT in PIMT. Additionally, single-photon emission computed tomography (SPECT) bone scans are frequently performed during the staging of lung cancers, and also for the indirect confirmation of lung cancer that has metastasized to the bone. The present study retrospectively analyzed the CT, ^18^F-FDG PET/CT and SPECT bone scan findings of PIMT occurring in patients >40 years old.

## Patients and methods

### Patient characteristics

Between September 2004 and June 2013, 10 patients, consisting of eight males and two females aged between 41 and 65 years, with a mean age of 56 years, were pathologically diagnosed with PIMT following surgical resection or a biopsy at Jinling Hospital, School of Medicine, Nanjing University (Nanjing, China). Of the 10 patients, eight underwent CT and two underwent ^18^F-FDG PET/CT. In four of the 10 patients, a SPECT bone scan using ^99m^Tc-methylene diphosphonate (MDP) was performed in order to determine the presence or absence of bone metastasis. The clinical data of the patients were retrospectively analyzed (Table I).

### CT examination

Of the eight patients who underwent CT examination, seven underwent plain and contrast-enhanced chest CT images and one underwent non-enhanced CT images alone. The scans were performed using a dual-source CT (Somatom Definition; Siemens Healthcare, Malvern, PA, USA) and a double-slice spiral CT scanners (Somatom Spirit; Siemens Healthcare). The CT parameters were as follows: A tube voltage of 120 kVp, a tube current of 150 mAs, a reconstruction interval of 2 mm, a slice thickness of 2 mm, a field of view of 250–350 mm and a matrix size of 512×512. The contrast-enhanced CT scan was performed with an intravenous injection of 100 ml iopamidol or 80 ml omnipaque at a rate of 2.5 ml/s, administered by a high-pressure autoinjector. CT enhancement was obtained in the arterial and venous phases, 20 and 50 sec after the injection of the contrast agent, respectively. The chest CT images were evaluated by the consensus of two experienced radiologists for the location, shape, size, density, margin and contrast enhancement of the lesions.

### ^18^F-FDG PET/CT imaging

The patients were asked to fast for at least 6 h prior to receiving an intravenous injection of ~370 MBq ^18^F-FDG. In addition, blood glucose was measured prior to the injection to ensure that levels were <140 mg/dl. The initial whole-body scan was carried out 60 min subsequent to the injection using a PET/CT system (Biography Sensation 16; Siemens Healthcare). The delayed scan was then localized to the lung and performed 120 min subsequent to the injection. The PET emission scan was performed with an acquisition time of 3 min for each bed. Next, PET data were obtained with the attenuation correction calculated from the coregistered CT images. Consequently, PET, CT and fused images of the early scan were displayed, in addition to those of the delayed imaging. The images were then visually interpreted by the consensus of two experienced nuclear medicine physicians for the location, shape, size, density, margin and ^18^F-FDG uptake pattern of the pulmonary lesions. The maximal standard uptake values (SUV_max_) of the dual-time-point were also calculated.

### SPECT bone scan

In four of the 10 patients, a SPECT bone scan (e.cam Signature Series; Siemens Healthcare) was performed 3 h subsequent to the intravenous administration of 1110 MBq ^99m^Tc-MDP. Anterior and posterior whole body planar images were acquired in a continuous mode at a scan speed of 20 cm/min using parallel-hole, low-energy, high-resolution collimators, with the patient in the supine position. The matrix size was 256×1024, and the zoom was 1.0 during the total acquisition. The whole body planar images were visually assessed by two experienced nuclear medicine physicians for the presence or absence of bone metastasis.

## Results

### CT findings

**Figure 1:**
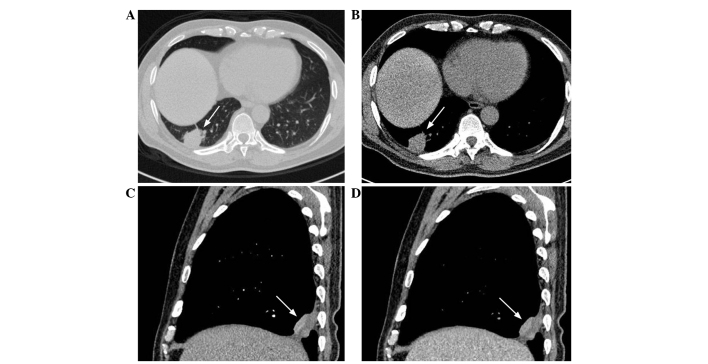
Plain chest computed tomography (CT) of a 54-year-old male with a pulmonary inflammatory myofibroblastic tumor. (A) Lung window setting and (B) mediastinum window setting revealing a well-defined, sub-pleural mass in the peripheral parenchyma of the right lower lobe. Contrast-enhanced CT images of the sagittal section in (C) the arterial phase and (D) the venous phase revealing high contrast enhancement in the solid portion of the mass, associated with the necrotic region.

**Figure 2:**
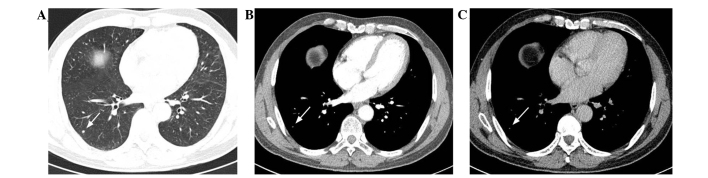
Plain chest computed tomography (CT) of a 58-year-old male with a pulmonary inflammatory myofibroblastic tumor. (A) Lung window setting revealing a small nodule with a maximum diameter of 5 mm located in the peripheral parenchyma of the right lower lobe. Due to its small size, contrast-enhanced CT images in (B) the arterial phase and (C) the venous phase were unable to reveal the degree of contrast enhancement of the nodule.

**Figure 3:**
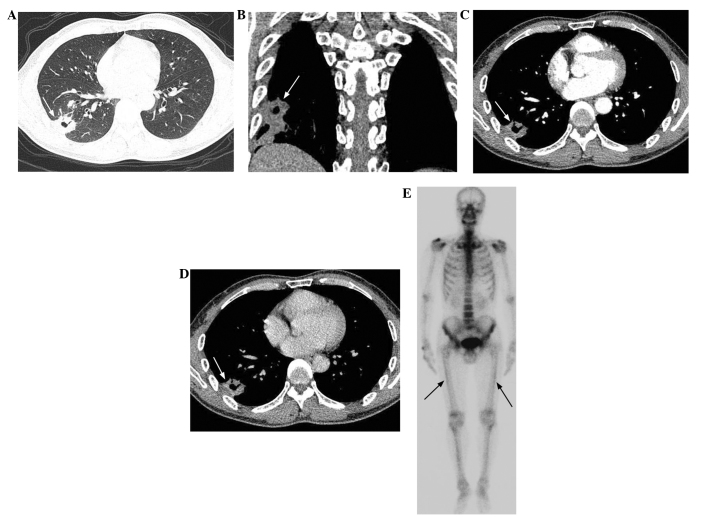
Plain chest computed tomography (CT) of a 56-year-old male with a pulmonary inflammatory myofibroblastic tumor. (A) Lung window setting revealing an ill-defined, sub-pleural mass in the peripheral parenchyma of the right lower lobe, associated with cavity and air bronchogram. The coronal CT scan in (B) the mediastinum window setting revealing the irregularly-shaped lesion. Contrast-enhanced CT scan in (C) the arterial phase and (D) the venous phase revealing the lesion with high contrast enhancement. (E) Single-photon emission computed tomography bone scan revealing bilateral atypical hypertrophic pulmonary osteoarthropathy in the cortex of the femurs.

**Figure 4:**
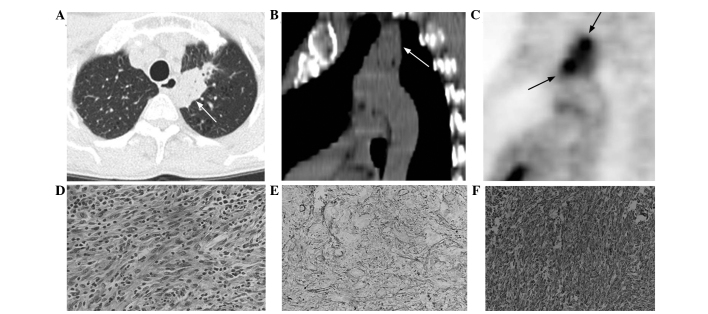
Plain chest computed tomography (CT) of a 59-year-old male with a pulmonary inflammatory myofibroblastic tumor. (A) Lung window setting revealing an ill-defined, sub-pleural mass in the peripheral parenchyma of the left upper lobe, associated with air bronchogram. Initial (B) CT and (C) positron emission tomography of the sagittal section revealing the lesion with increased heterogeneous tracer uptake. (D) Microscope image stained with hematoxylin and eosin revealing the proliferation of spindled fibroblastic myofibroblastic cells in fascicles, accompanied by an inflammatory infiltrate of lymphocytes and plasma cells. Immunohistochemical images revealing positive staining with (E) smooth muscle actin and (F) vimentin.

The CT findings of PIMT in the present study were based on 10 patients, consisting of eight who underwent routine CT examination and two who underwent the unenhanced CT component of PET/CT (Table II). CT revealed 10 lesions, of which four were located in the upper lobe and six were located in the lower lobe (Fig. 1A). In total, three lesions were located in the left lung and seven involved the right lung. A central parenchymal lesion was only identified in one patient, but the presence of a peripheral parenchymal lesion was revealed in nine patients, of which six presented with a sub-pleural mass (Fig. 1B). The maximum diameters of the ten lesions, comprising eight masses and two nodules, ranged between 5 and 57 mm. The lesions were either well- (n=4; Fig. 2A) or ill-defined (n=6; Fig. 3A) and round to oval (n=5; Fig. 2A) or irregular (n=5; Fig. 4A and B) in shape. The associated CT findings demonstrated calcification (n=3), necrosis (n=6; Fig. 1C and D), cavity (n=4; Fig. 3A and B), air bronchogram (n=6; Fig. 4A and B) and obstructive pneumonia (n=1). In four of the six lesions with necrosis, peripheral necrosis was evident within the lesions (Fig. 1C and D).

In total, seven patients underwent contrast-enhanced CT, but one pulmonary lesion was unable to be evaluated by contrast enhancement due to a small maximum diameter of 5 mm (Fig. 2B and C). The degree of contrast enhancement was measured in six patients. The results revealed that lesions increased in attenuation by between 12 and 79.1 Hounsfield units (HU; mean, 38.5±22.8 HU) in the arterial phase, and between 30.4 and 57.9 HU (mean, 44.2±10.2 HU) in the venous phase (Fig. 3C and D).

### ^18^F-FDG PET/CT findings

In total, two patients with PIMT underwent ^18^F-FDG PET/CT imaging. In one patient, a mass-like high FDG uptake was identified in the left lower lobe. The homogeneous radioactive uptake following early and delayed imaging exhibited a SUV_max_ of 6.0 and 6.9, respectively. The SUV_max_ obtained from the delayed imaging exhibited a 15% increase compared with the early imaging. A heterogeneous elevated tracer uptake in the left upper lobe was observed in the other patient. In total, two nodule-like radioactive foci were identified within this lesion (Fig. 4C). The nodules exhibited an early SUV_max_ of 5.4 and a delayed SUV_max_ of 5.9, an increase of 9%. The PET/CT imaging characteristics are presented in Table III.

### Bone scan findings

The patients that underwent SPECT were initially suspected of having lung cancer prior to the surgery, which was determined by the absence of bone metastases. Of the four patients, two demonstrated no abnormal whole-body bone tracer uptake. Overall, one patient demonstrated a mildly increased tracer uptake, with a bilateral linear distribution along the cortex of the femurs, which was suggestive of atypical hypertrophic pulmonary osteoarthropathy (HPO) (Fig. 3E). The other patient possessed three foci that were located in the ribs, which were considered to demonstrate non-specific uptake in combination with the corresponding CT images.

### Pathology and immunohistochemistry

Microscopic analysis revealed that the tumors were composed of bundles of spindle cells, including myofibroblasts and fibroblasts, arranged in a fascicular or storiform manner, and surrounded by chronic inflammatory cell infiltration (Fig. 4D). These inflammatory cells were primarily composed of plasma cells, lymphocytes and granulocytes. Specimens obtained from the five patients were subjected to immunohistochemical examination. Positive staining for smooth muscle actin (Fig. 4E) was identified in all five cases, vimentin (Fig. 4F) in two cases, and cluster of differentiation (CD)68, CD34, CD20 and CD3 in one case.

## Discussion

PIMT is extremely uncommon. Overall, it is reported that ~40% of all cases occur in adults >40 years old (15) who are more likely to be affected by lung cancers. For this reason, a considerable number of PIMTs occurring in this age group are misdiagnosed as lung cancer prior to a biopsy or surgical resection (15,16). In the present study, which included ten patients >40 years old with PIMT, eight were originally suspected of having lung cancer prior to a wedge resection or lobectomy. In general, PIMT affects males and females equally (5,7,16,17). However, there was a significant male predilection (8/10) in the present study, which could be attributed to the older age group included. Patients with PIMT are usually asymptomatic, or present with non-specific symptoms, including coughing, hemoptysis, dyspnea, fever and chest pain (16,18,19). In the present study, two patients (2/10) were asymptomatic, and one pulmonary lesion was detected incidentally on a routine health check-up. Although the etiology of PIMT remains unknown, it has been hypothesized that pulmonary infection may contribute to the pathogenesis of the disorder, as prior pulmonary disease has only been reported in 30% of patients (14,16). The present study of 10 patients included only one patient with a previous pulmonary disease, but included six patients with a history of smoking, which indicates that smoking may be a factor that contributes to PIMT.

According to a study by Kakitsubata *et al* (16), no significant differences were identified between PIMT involving the left or right side. However, PIMT does exhibit a predilection for the lower lobes. In the present study, the right (4/10) and left lower lobes (2/10) were more frequently affected by PIMT compared with the right (3/10) and left (1/10) upper lobes. In addition, lesions located in the peripheral parenchyma (9/10) and sub-pleura (6/10) were observed more often, which indicates that PIMT may also have a predilection for these lung regions. It has been reported that the size of PIMT usually ranges between 10 and 150 mm (14,16,20). With the exception of one case, the size of the lesions in the present study corresponded with those previously reported. The exception in the present study had a maximum diameter of 5 mm. To the best of our knowledge, this is the smallest reported PIMT, which may aid in understanding the nature of PIMT.

At present, CT is the most widely used method for the detection and differentiation of pulmonary masses, including lung cancer, tuberculosis, inflammatory pseudotumors and PIMT (21). However, due to its rarity, major studies concerning CT delineations of PIMT are in the form of case studies. In total, two previous studies have included only ~10 cases (15,16). Although classic PIMT is defined as a slow-growing, solitary, round to oval-shaped and well-circumscribed mass in the peripheral regions of the lower lobes (16), CT manifestations of PIMT are generally diverse. A number of studies have reported that PIMTs present as ill-defined or irregular lesions (6,16,22–24). In addition, other CT findings, including calcification, cavity, necrosis, obstructive atelectasis and pneumonia have also been reported in several previous studies (8,9,15,16,18,20). According to the results of Kakitsubata *et al* (16), the incidence of calcification and cavities in cases of PIMT range between 4 and 17.5%, and 50 and 57%, respectively. The results of the present study are partly in agreement with those of previous studies. In total, four cases included in the present study were consistent with classic PIMT, and the other six cases existed as ill-defined or irregular masses. The presence of calcification and cavities was 30 (3/10) and 40% (4/10), respectively. However, necrosis and air bronchogram (60%; 6/10) were relatively frequent, which are factors rarely mentioned in previous studies.

The majority of PIMT cases reported in previous studies have demonstrated homogeneous or heterogeneous CT contrast enhancement (15,16,19,21). In addition, the degree of enhancement has varied between them. Calabrese *et al* (25) reported two cases of PIMT with mild increases in density following enhancement. Furthermore, Kim *et al* (15) assessed the degree of contrast enhancement in seven lesions and identified an increase in attenuation by 13–89 HU following contrast administration. Chen *et al* (19) described a patient with PIMT that demonstrated weak enhancement in the arterial phase. According to the study by Takayama *et al* (12), delayed enhancement was evident in two cases of PIMT. In this study, early-phase images with slight enhancement and delayed-phase images with heterogeneous enhancement were obtained 70 and 300 sec after the injection of the contrast medium, respectively. The presence of delayed enhancement has also been confirmed in certain studies reporting IMT occurring at other sites, including the heart, liver, kidney and the greater omentum (26–30). However, there have been certain cases of PIMT occurring in the absence of contrast enhancement, as reported by Kakitsubata *et al* (16) and Dhouib *et al* (14). The present study evaluated the degree of contrast enhancement in six patients with PIMT, which included the arterial and venous phases at 20 and 50 sec after the injection of the contrast agent, respectively. All lesions demonstrated moderate to high contrast enhancement in each phase, and four exhibited delayed enhancement.

To date, there have been a number of studies concerning ^18^F-FDG uptake in PIMT (5,6,9,14,19,25,31,32). These studies all demonstrated increased ^18^F-FDG uptake, with the SUV values ranging between 2.8 and 25. The present study included two cases of PIMT that were analyzed by ^18^F-FDG PET/CT, and exhibited elevated ^18^F-FDG uptake with SUV values above the normal range. Although ^18^F-FDG is known to accumulate in a number of malignancies, including lung cancer, it has also been observed to actively concentrate in certain benign pulmonary diseases, such as pneumonia, Wegener’s granulomatosis, tuberculosis, fungal infections and abscesses (32). Overall, two cases in the present study were not identified as having lung cancer following PET/CT, which confirmed the presence of pulmonary unifocal lesions without metastatic disease. Furthermore, the two PIMT lesions underwent delayed PET/CT imaging, which had not been performed in previous case studies. In the two cases, the SUV_max_ of the delayed imaging were higher than those of the initial imaging. A number of previous studies have revealed that dual time-point ^18^F-FDG PET or PET/CT imaging may aid in distinguishing malignant from benign processes (33–37). Malignant diseases, including hepatocellular carcinoma, pancreatic cancer, lung cancer and malignant lymphoma have demonstrated increased ^18^F-FDG accumulation on delayed images compared with early images. However, there have been other studies that have identified a significant overlap in ^18^F-FDG uptake patterns between benign and malignant lesions, particularly for pulmonary lesions, and even on delayed time-point images (38–42). As revealed by the present study, an increased delayed imaging SUV_max_ of the lung cannot guarantee the presence of malignancy. These ^18^F-FDG PET/CT findings may aid in the diagnosis of PIMT, but further studies that include larger patient populations are required in order to expand these results.

The bone scan features of PIMT have not been depicted in previous studies. The present study included four patients who underwent SPECT for the detection of bone metastases. Although there were no definitive bone metastases detected, SPECT revealed atypical HPO in one PIMT case. HPO can occur secondary to various neoplastic and non-neoplastic diseases, including primary lung cancer, metastatic pulmonary disease and cystic fibrosis (43). The ‘tram-line’ or ‘double-stripe’ sign, which represents abnormal periosteal bone formation, is the classic appearance of HPO upon bone scanning (43,44). With the exception of PIMT, CT did not identify any other pulmonary diseases in the patient with HPO in the present study. Based upon the chest CT findings, it was hypothesized that the HPO detected by SPECT was caused by the PIMT. To the best of our knowledge, this is the first study to demonstrate that PIMT can lead to HPO.

PIMT occurring in patients >40 years old is extremely rare and the symptoms often mimic those of lung cancer (13,19). Certain imaging features are relatively common in PIMT patients of this age group, such as being located in the lower lobe and peripheral parenchyma, necrosis, air bronchogram, moderate to high contrast enhancement or delayed enhancement, increased ^18^F-FDG uptake with an elevated SUV_max_ upon delayed imaging, and the absence of definitive metastases. Although these imaging features remain non-specific for the distinction between PIMT and lung cancer, they may aid in enhancing the awareness of PIMT during the differential diagnosis of lung masses. The combination of imaging modalities, including CT, ^18^F-FDG PET/CT and SPECT bone scans, may aid in successfully diagnosing PIMT, determining the extent of the tumor and also managing the treatment.

## Figures and Tables

**Table I:** Clinical data of 10 cases of pulmonary inflammatory myofibroblastic tumor.

Patient	Gender	Age, years	Clinical symptoms	Medical history	Surgical management
1	M	54	Cough, expectoration, chest pain	Smoking, hypertension	Wedge resection
2	M	62	Cough, expectoration, hemoptysis	Bronchiectasis, smoking	Lobectomy
3	M	56	None	None	Lobectomy
4	M	56	Cough, expectoration, hemoptysis	Smoking	Lobectomy
5	F	54	None	Systemic lupus erythematosus	Lobectomy
6	F	65	Cough	Hypertension	Lobectomy
7	M	58	Chest pain	Hypertension	Lobectomy
8	M	49	Cough, fever, chest pain	Smoking	Percutaneous biopsy
9	M	41	Back pain, fever	Smoking	Percutaneous biopsy
10	M	59	Cough, expectoration, fever	Smoking	Lobectomy

M, male; F, female.

**Table II:** Computed tomograghy findings of eight cases of pulmonary inflammatory myofibroblastic tumor.

Patient	Location	Size, mm	Margin	Shape	AP NCE	VP NCE	Others
1	RLL, PP, SP	44	Well-defined	Oval	37.7	34.0	Calcification, necrosis
2	LLL, PP	34	Ill-defined	Round	12.0	30.4	Necrosis, cavity
3	RLL, PP, SP	43	Ill-defined	Irregular	79.1	48.0	Calcification, cavity, air bronchogram
4	RUL, PP, SP	38	Ill-defined	Irregular	44.2	57.9	Cavity, necrosis, air bronchogram
5	RLL, PP	32	Ill-defined	Irregular	32.5	47.0	Necrosis, air bronchogram
6	RUL, PP	30	Ill-defined	Irregular	25.3	47.8	Cavity, necrosis, air bronchogram
7	RLL, PP	5	Well-defined	Round	NA	NA	
8	RUL, CP	26	Well-defined	Oval			Necrosis, obstructive pneumonia

RLL, right lower lobe; LLL, left lower lobe; RUL, right upper lobe; CP, central parenchyma; PP, peripheral parenchyma; SP, Sub-pleural; NCE, net contrast enhancement; AP, arterial phase; VP, venous phase; NA, not available.

**Table III:** ^18^F-fluorodeoxyglucose positron emisission tomography/computed tomography findings of two cases of pulmonary inflammatory myofibroblastic tumor.

Pt. No.	Location	Size, mm	Margin	Shape	Others	UP	EI SUV_max_	DI SUV_max_
9	LLL, PP, SP	52	Well-defined	Oval	Air bronchogram	Homogeneous	6.0	6.9
10	LUL, PP, SP	57	Ill-defined	Irregular	Calcification, air bronchogram	Heterogeneous	5.4	5.9

Pt, patient; No, number; LLL, left lower lobe; LUL, left upper lobe; PP, peripheral parenchyma; SP, sub-pleural; UP, uptake pattern; EI, early imaging; DI, delayed imaging; SUV_max_; maximal standard uptake value.
